# Diagnostic Role and Clinical Impact of Zr-Girentuximab PET-CT for the Diagnosis and Treatment of Clear-Cell Renal Cell Carcinoma

**DOI:** 10.3390/diagnostics16091323

**Published:** 2026-04-28

**Authors:** Daniel A. González-Padilla, Felipe Villacampa-Auba, Jorge Caño-Velasco, José Daniel Subiela, María Rodríguez, Carlos Yánez, Andrés Calva, Vanessa Talavera, Carmina Muñoz, Marcos Torres, Guillermo Barbas, Guillermo Andrés, Daniel Sánchez-Zalabardo, Edgar Fernando Guillén-Valderrama, Bernardino Miñana-López

**Affiliations:** 1 Department of Urology, Kidney and Bladder Cancer Unit, Clínica Universidad de Navarra, 28027 Madrid, Spain; dgonzalezp@unav.es (D.A.G.-P.);; 2 Department of Urology, Kidney and Bladder Cancer Unit, Clínica Universidad de Navarra, 31008 Pamplona, Spain; 3 Department of Urology, Hospital Universitario Ramón y Cajal, Instituto Ramón y Cajal de Investigación Sanitaria (IRYCIS), Universidad de Alcalá, 28034 Madrid, Spain; 4 Department of Nuclear Medicine, Clínica Universidad de Navarra, 28027 Madrid, Spain

**Keywords:** girentuximab, PET-CT, renal cell carcinoma, carbonic anhydrase IX, ZIRCON

## Abstract

**Background/Objectives:** Clear-cell renal cell carcinoma (ccRCC) represents the predominant histologic subtype of renal cancer and poses persistent diagnostic challenges, particularly in the evaluation of small renal masses, where conventional imaging and biopsy have relevant limitations. Molecular imaging targeting carbonic anhydrase IX (CAIX) has emerged as a promising non-invasive alternative. This narrative review aims to summarize the biological rationale, diagnostic performance, and potential clinical applications of [^89^Zr]Zr-girentuximab positron emission tomography-computed tomography (girentuximab PET-CT) in ccRCC, as well as to discuss its current limitations and future directions. **Methods:** A narrative synthesis of published phase 1–3 clinical trials, post hoc analyses, and early clinical series evaluating girentuximab PET-CT was performed, focusing on diagnostic accuracy, clinical impact in localized and metastatic disease, and emerging theranostic applications. **Results:** The phase 3 ZIRCON trial demonstrated high diagnostic accuracy of girentuximab PET-CT for indeterminate renal masses ≤7 cm, with a sensitivity of 85% and specificity of 87%, as well as performance exceeding 96% for lesions <2 cm. Early studies suggest that this modality may influence clinical decision-making by supporting active surveillance, avoiding biopsy, and refining surgical or ablative strategies, although evidence remains limited by small cohorts and lack of long-term outcome data. Exploratory data indicate improved lesion detection in metastatic ccRCC, but the absence of systematic histopathologic confirmation restricts routine staging use. **Conclusions:** Girentuximab PET-CT is a highly accurate, CAIX-targeted molecular imaging technique with the potential to transform the diagnostic pathway of ccRCC. While current evidence supports its use in selected localized settings, broader clinical adoption will require prospective validation of its impact on patient outcomes and management strategies.

## 1. Introduction

Renal cell carcinoma (RCC) is the most common malignancy of the kidney, with clear-cell renal cell carcinoma (ccRCC) accounting for approximately 70–80% of cases [1,2].

The incidental finding of small renal masses (SRMs) defined as a contrast-enhancing renal lesion that measures 4 cm or less in maximal diameter on imaging studies [3] has risen sharply in recent decades, with about 20% of these masses being ultimately benign [4,5].

Current conventional imaging, primarily contrast-enhanced computed tomography (CT) and Magnetic Resonance Imaging (MRI), relies on anatomical features and enhancement patterns to try to differentiate ccRCC from benign lesions such as oncocytoma or angiomyolipoma. While percutaneous biopsy may provide a histological diagnosis, it has a non-diagnostic rate from 8% up to 19% while carrying a non-negligible risk of complications of 2–11% [6,7,8].

All things considered, there is still a need for a highly accurate, non-invasive technique to characterize renal masses to avoid both unnecessary surgeries and unnecessary biopsies.

## 2. The Molecular Basis of Girentuximab and Carbonic Anhydrase IX

Girentuximab positron emission tomography (PET)-CT (^89^Zr-DFO-girentuximab or TLX250-CDx) is a novel imaging modality based on a chimeric monoclonal antibody (girentuximab) that binds with high affinity to carbonic anhydrase IX (CAIX) [9]. Approximately 95% of ccRCCs are characterized by inactivation of the Von Hippel–Lindau (VHL) tumor suppressor gene; VHL inactivation results in the stabilization and accumulation of the hypoxia-inducible factors, which in turn promotes overexpression of various target genes, including CAIX [10,11].

CAIX is a transmembrane glycoprotein that plays a critical role in intracellular pH regulation, allowing tumor cells to survive in hypoxic and acidic microenvironments. Importantly for imaging, CAIX is highly and selectively expressed on the surface of approximately 95% of ccRCC tumors, while being virtually absent in normal renal parenchyma, benign renal cysts, oncocytomas, and most non-ccRCC subtypes. This extreme differential expression provides the biological contrast necessary to reliably distinguish aggressive ccRCC from indolent or benign renal masses [12].

Girentuximab is a chimeric IgG1κ monoclonal antibody derived from the G250 clone and engineered to bind with high affinity to an extracellular epitope of CAIX. Following intravenous administration, girentuximab circulates systemically and selectively accumulates in CAIX-expressing tissue, exhibiting a highly specific “lock-and-key-type” binding mechanism while sparing healthy organs and non-clear-cell tumors. After binding to CAIX, the antibody–antigen complex undergoes cellular internalization, producing a sustained high-contrast PET signal [12].

Zirconium-89 (^89^Zr) serves as the positron-emitting radionuclide payload that is chemically conjugated to the chimeric monoclonal antibody (girentuximab) via desferrioxamine.

Desferrioxamine (DFO) is the chelator that stably links Zr to girentuximab, enabling safe and accurate PET imaging in ccRCC. By preventing radionuclide release and bone uptake, it preserves image quality, minimizes radiation toxicity, and supports reliable intracellular tracer retention, which is essential for the diagnostic performance of girentuximab PET-CT [13].

The resulting radiotracer, ^89^Zr-DFO-girentuximab, selectively illuminates CAIX-expressing ccRCC upon PET-CT imaging.

### 2.1. Image Acquisition Protocol

The diagnostic protocol involves a single intravenous administration of 37 MBq (±10%) of Zr-DFO-girentuximab, utilizing a 10 mg antibody mass dose optimized to maximize tumor-to-background contrast; Zirconium-89 is selected for its 78.4 h half-life, which matches antibody kinetics and provides favorable tumor retention and spatial resolution.

After administration on Day 0 and short post-injection monitoring, imaging is intentionally delayed to approximately five days (range: 3 to 7 days) to allow blood-pool clearance and tumor uptake, during which patients may resume normal activities with minimal radiation precautions.

**Figure 1:**
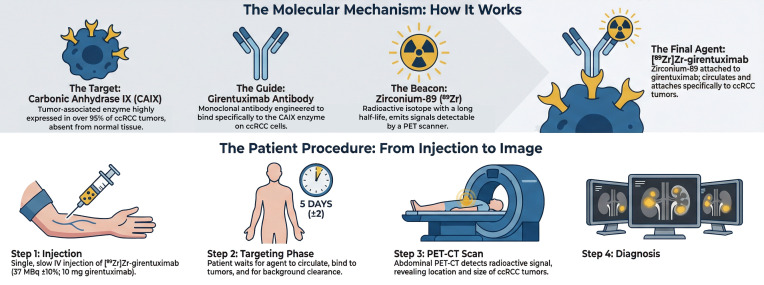
Graphical representation of the mechanism of girentuximab PET-CT, the procedure and its diagnostic performance. ccRCC = clear-cell renal cell carcinoma; CAIX = carbonic anhydrase IX; PET = positron emission tomography; IV = intravenous; PET-CT = positron emission tomography-computed tomography.

Image acquisition consists of a hybrid PET and low-dose CT scan; hepatobiliary, rather than urinary clearance, minimizes renal collecting system activity, enabling clear visualization of renal cortical lesions. Interpretation is based on visual and quantitative tracer uptake, with positive scans showing uptake above background [9]. This process is summarized in Figure 1.

### 2.2. Diagnostic Performance of Girentuximab PET-CT

The landmark trial studying girentuximab PET-CT was the ZIRCON trial. This was a prospective, open-label, multicenter, phase 3 study involving patients scheduled for radical or partial nephrectomy of an indeterminate renal mass 7 cm or smaller (cT1), where ccRCC was suspected. The co-primary endpoints were the sensitivity and specificity of the girentuximab PET-CT scan to detect ccRCC, using histopathological analysis of the surgical specimen as the gold standard [9].

A total of 284 evaluable patients were included in the primary analysis. The results, as determined by a blinded central review of three independent readers, showed a mean sensitivity of 85.5% (95% CI [confidence interval] 81.5–89.6) and mean specificity of 87.0% (95% CI 81.0–93.1); the full diagnostic performance is summarized in Table 1. As a comparison, we can consider the diagnostic performance of PSMA (prostate-specific membrane antigen) PET-CT in high-risk prostate cancer as described in the ProPSMA trial which showed a sensitivity of 85%, specificity of 98% and an overall accuracy of 92% [14].

### 2.3. Comparison with Other Imaging Technologies

While the results of the ZIRCON trial were outstanding, the conventional imaging already available in daily practice has a moderately good performance.

Conventional CT sensitivity for the diagnosis of localized RCC is approximately 88% and specificity is 75%. Contrast-enhanced CT is the standard modality for initial tumor characterization and staging, but its main limitations are that it cannot reliably differentiate benign from malignant renal masses (e.g., ccRCC from oncocytoma); girentuximab PET-CT can add certainty when it comes to ccRCC suspicion [15,16]. MRI has shown sensitivity for the diagnosis of primary renal cell carcinoma that ranges from 80% to 88%, with a specificity of 89–90%; MRI is particularly useful for indeterminate small renal masses on CT (masses smaller than 15 mm of size), patients with contraindication to iodinated contrast, assessment of complex cystic masses, and for assessing venous invasion; out of all these scenarios, girentuximab PET-CT can only provide additional information in solid renal masses under 15 mm in size, as the ZIRCON trial did not include patients with cystic renal masses, renal insufficiency or ≥T2 [9,15,16,17]. Fluorodeoxyglucose (FDG) PET-CT for the diagnosis of primary renal cell carcinoma has a sensitivity of 46–60% and specificity of 66–100%. FDG PET-CT is not recommended for routine staging of localized renal cell carcinoma because of its limited sensitivity, largely due to physiologic renal excretion of FDG. However, for the detection of distant metastases—particularly osseous and pulmonary lesions—both sensitivity and specificity increase substantially, reaching 86–94% for sensitivity and 88–100% for specificity; in this case, FDG PET-CT and girentuximab PET-CT are mutually complementary as FDG PET-CT cannot appropriately evaluate renal masses and girentuximab is not validated for the evaluation of distant metastasis [17,18,19,20,21,22]. Then, ^99^Tc-sestamibi SPECT/CT (Sestamibi) uses mitochondrial density to distinguish benign oncocytic lesions (“hot”) from aggressive renal cell carcinomas (“cold”); Sestamibi has a pooled sensitivity of 89–92% and specificity of 88–91% [23]. Crucially, its specificity for excluding clear-cell and papillary renal cell carcinoma reaches 98% [23] In this regard, Sestamibi is complementary to girentuximab PET-CT, as Sestamibi is particularly useful for ruling in oncocytomas (when “hot”) and girentuximab PET-CT is particularly useful for ruling in ccRCC (when positive). Finally, contrast-enhanced ultrasound (CEUS) is a valuable adjunct for evaluating small renal masses, particularly those smaller than 3 cm, demonstrating high diagnostic accuracy with reported sensitivity of approximately 88–100% and specificity of 80–97% for distinguishing malignant from benign lesions. It is especially effective in differentiating ccRCC from angiomyolipoma and oncocytoma using characteristic enhancement features such as arterial-phase hypoenhancement, rapid wash-out, and perilesional rim enhancement, some of which show specificity approaching 95–100%. CEUS offers superior contrast resolution for detecting minimal vascularity, improves characterization of cystic lesions and Bosniak classification compared with CT or MRI, and allows quantitative perfusion analysis to further refine lesion subtyping. Importantly, CEUS avoids ionizing radiation and nephrotoxic contrast, making it particularly suitable for patients with renal function impairment; nonetheless, CEUS is highly operator-dependent and has not undergone scrutiny under large-scale randomized trials, hence the validity of the results may not be transferable to most centers around the world [24,25]. All things considered, girentuximab PET-CT is the only imaging study that can reliably confirm the suspicion of ccRCC in a localized renal mass while other imaging studies provide other useful information. These characteristics are summarized in Table 2.

### 2.4. Clinical Applications in Localized Renal Cell Carcinoma

A study by Hekman et al. (ZIRDEE trial) used girentuximab PET-CT in 16 patients with “indistinct” primary renal masses. Six patients had a positive girentuximab PET-CT, five of them underwent surgery, and all of them were ccRCC; the sixth patient had VHL syndrome and the girentuximab PET-CT showed another renal mass occult to MRI, so this patient underwent percutaneous cryoablation of the largest mass. Nine patients where girentuximab PET-CT-negative and those patients were managed with active surveillance with no signs of progression after a mean follow-up of 13 months [26]. This study showed the potential of girentuximab PET-CT as a tool to help in complex decisions and may help tip the scale towards active surveillance or surgical treatment in some scenarios, yet its findings are limited by its small size, the lack of universal pathological correlation, and the relatively short follow-up for surveillance cohorts.

A post hoc analysis of the ZIRCON trial, called ZIRCON-X, was presented at the 2025 SUO (Society of Urological Oncology) meeting. In this study the authors used the information gathered from the original ZIRCON trial and simulated a multi-disciplinary team discussion with four different teams at different institutions. The authors analyzed the decision taken with conventional imaging and the changes in management with girentuximab PET-CT images available; the results reported were that over one third of all patients would have had a major change in management and approximately 30% of patients had a treatment escalation or de-escalation, supporting the potential use of this imaging study to further refine the diagnostic and therapeutic pathway in these patients [27]. This study is limited by its post hoc non-interventional design, as all decisions were hypothetical rather than implemented management changes, which has an inherent bias; as management decisions were not executed, data on actual oncological outcomes are absent.

In another clinical scenario, González-Padilla et al. published a case series of three patients with prior radical nephrectomies with ccRCC in the past with de novo small renal masses in their solitary kidney. Girentuximab PET-CT helped in decision-making and avoiding biopsies in these high-risk patients; in two of them, girentuximab PET-CT was able to detect additional masses that were not seen in conventional imaging, whereas in the third patient it helped ruling out ccRCC in a Bosniak IV cyst. The former patients were managed with percutaneous ablation and the latter with active surveillance [28].

Nonetheless, it is important to emphasize that while the diagnostic performance of girentuximab PET-CT is good, it has only been compared with final pathology of patients undergoing surgery, hence no head-to-head comparison with other imaging modalities has been performed; therefore, there is no clear evidence-based indication girentuximab PET-CT over other imaging modalities.

## 3. Role in Metastatic Renal Cell Carcinoma and Prognostic Assessment

The utility of girentuximab PET-CT beyond primary diagnosis may involve the assessment of metastatic ccRCC as suggested from the IMPACT-RCC study by Verhoeff et al. In this study the authors compared imaging modalities in 42 newly diagnosed metastatic ccRCC patients; patients underwent conventional CT scanning, girentuximab PET-CT and FDG-PET-CT at baseline. Scans were independently reviewed and lesions of ≥10 mm and lymph nodes of ≥15 mm at CT were analyzed [29].

Authors found 449 lesions (median of seven per patient) of which 42%, 22% and 10% were in the lung, lymph nodes and bone respectively. The combination of girentuximab PET-CT and conventional CT detected 91% of lesions versus 56% by conventional CT alone, while the combination of FDG PET-CT found 84% of lesions.

The most obvious limitation of this study is the lack of histopathological confirmation of these lesions (no biopsy and potential false-positives); also, to avoid overestimating disease burden, the study applied strict diameter cut-offs for CT (10 mm for lung and bone lesions and 15 mm for lymph nodes) by excluding smaller but potentially suspicious lesions on CT while allowing “visually positive” PET-CT findings regardless of size; the study likely underestimated the baseline performance of conventional CT scan.

Additionally, similarly to the paradigm with PSMA PET-CT versus conventional imaging in prostate cancer, it becomes a philosophical question of whether patients already considered metastatic by conventional CT benefit from finding additional metastasis in PET-CT, considering that only patients with suspected metastasis were included in this study. Further research into patients of unknown metastatic status with histopathologic or temporal confirmation will be needed before implementing girentuximab PET-CT for this purpose.

### 3.1. Potential Clinical Applications

Girentuximab PET-CT has emerged as a highly accurate, non-invasive imaging modality for the study of renal masses; while this advancement provides potentially superior diagnostic performance compared to its imaging predecessors (conventional CT, MRI, FDG PET-CT, Sestamibi, and CEUS) when it comes to diagnosing ccRCC in small renal masses, and with an equivalent diagnostic performance to a percutaneous biopsy without the inherent risks of this invasive procedure, there is still no clear consensus as to when it would be indicated to perform this imaging study. We propose the following potential indications:

- Avoiding percutaneous kidney biopsies in patients in whom a serious complication is particularly undesired (kidney transplant recipients, solitary kidneys, and anticoagulated patients);
- Supporting shared decision-making among patients with small renal masses considering active surveillance as an option, in patients with a negative girentuximab PET-TC it could tip the scale towards surveillance;
- Follow-up of patients with VHL syndrome, considering the higher accuracy of this imaging study in smaller lesions;
- Follow-up of patients after percutaneous ablation of SRMs, in whom recurrences could be potentially diagnosed earlier by girentuximab PET-CT.

It is important to make clear that none of these indications have been validated in clinical trials, and they should be considered only for investigational purposes.

### 3.2. Limitations

Some limitations of the girentuximab PET-CT are that the expression of CAIX is limited to clear-cell histology, hence a negative PET-CT can only tell with an 86% precision that the renal mass is not clear-cell but remains uninformative on whether it is clearly benign.

Another limitation is the logistical complexity and surrounding costs to the healthcare systems and financial toxicity to the patients, requiring two separate visits to the hospital (one for injection and one for the PET-CT imaging itself), using additional resources when compared to other types of PET-CTs.

Radiation exposure is another limitation to be considered, as girentuximab PET-CT delivers approximately three times the radiation of a PSMA PET-CT (20–30 mSv vs. 6–14 mSv) and twice the radiation of an FDG PET-CT (20–30 mSv vs. 10–17 mSv) [9].

While girentuximab PET-CT has shown a high sensitivity and specificity to diagnose localized ccRCC, it remains to be determined in prospective studies whether these results truly change management, whether it can increase active surveillance or reduce invasive interventions without a percutaneous biopsy, and preserve quality of life without impacting cancer-specific survival.

## 4. Future Directions

Future studies should evaluate whether girentuximab PET-CT can effectively differentiate benign from malignant cystic renal masses (Bosniak IIF, III and IV) which can harbor cancer in 46% to 84% of cases when using the 2019 Bosniak classification [30]. Another potential indication is the clinical staging in ccRCC (for the detection of metastatic disease); both of these will require independent clinical trials.

Ongoing studies are evaluating the potential role of girentuximab as a therapeutic agent focused primarily on its conjugation with the beta-particle-emitting isotope lutetium-177 (^177^Lu). There are currently three ongoing studies in this space:

- The STARLITE-1 trial (NCT05663710) is a phase 1b/2, investigator-initiated study led by MD Anderson Cancer Center which explores whether targeted radioimmunotherapy can be safely and effectively integrated into first-line treatment for advanced ccRCC, with the goal of meaningfully improving deep response rates. STARLITE-1 is testing the hypothesis that the addition of CAIX-targeted radiation can substantially increase complete response rates, with a predefined target of 18%. The trial leverages the near-universal overexpression of CAIX in ccRCC by employing ^177^Lu-girentuximab, which delivers localized β-particle radiation directly to tumor cells. Beyond cytotoxicity, radiation-induced DNA damage is hypothesized to activate the cGAS–STING pathway, a central driver of innate immune signaling. This activation may enhance T-cell priming and infiltration into the tumor microenvironment, thereby potentiating the antitumor activity of other therapies (in this case nivolumab and cabozantinib).

STARLITE-1 plans to enroll up to 100 treatment-naïve patients with biopsy-confirmed metastatic ccRCC. A five-patient safety lead-in was incorporated to specifically assess hematologic toxicity, historically a limiting factor for radiolabeled girentuximab. Patients receive ^177^Lu-girentuximab at 1480 MBq/m^2^—approximately 61% of the established single-agent maximum tolerated dose—administered every 12 weeks for up to three cycles. Standard-dose nivolumab and cabozantinib are introduced starting in cycle 2, forming a triplet regimen designed to balance efficacy and tolerability. The primary endpoint is the achievement of an 18% complete response rate. Secondary endpoints include objective response rate, progression-free survival per RECIST, and overall survival. Importantly, the trial incorporates translational imaging with ^18^F-F-AraG PET, enabling non-invasive visualization of activated T-cell infiltration before and after treatment, thereby directly testing the proposed immunologic mechanism. If successful, STARLITE-1 could establish CAIX-directed theranostics in advanced ccRCC [31].

STARLITE-2 trial (NCT05239533) is a prospective, phase 2 trial evaluating the safety and antitumor activity of a dual-agent regimen in pretreated ccRCC. In contrast to STARLITE-1, which enrolls treatment-naïve patients, this study focuses on individuals with advanced disease who have failed prior tyrosine kinase inhibitor and/or immune checkpoint inhibitor therapy. The investigational regimen combines ^177^Lu-girentuximab (TLX250), a CAIX-directed radioimmunotherapy delivering β-particle radiation to tumor cells, with nivolumab, a programmed death-1 (PD-1) inhibitor. A dose-escalation phase is incorporated to define the maximum tolerated dose of the radiopharmaceutical when administered concurrently with immunotherapy, a critical consideration in a heavily pretreated population. Historically, the clinical application of radioimmunotherapy with intact monoclonal antibodies has been constrained by hematologic toxicity, stemming from prolonged systemic circulation and bone marrow irradiation. STARLITE-2 directly addresses this challenge through careful dose escalation, fractionated administration, and close hematologic monitoring, aiming to optimize the therapeutic index without compromising efficacy [32].

LUTEON trial (NCT07197580) is the first phase 3 trial in the theranostic space of girentuximab; it is a randomized, multicenter, open-label trial comparing ^177^Lu-TLX250 (lutetium-177–girentuximab tetraxetan) with investigator’s choice of standard single-agent therapy. Eligible patients must have relapsed or experienced progressive disease after two to three prior lines of systemic therapy, including mandatory exposure to a PD-1 or PD-L1 inhibitor. Enrollment requires confirmed CAIX expression, typically established through prior molecular imaging with ^89^Zr-girentuximab PET/CT as validated in the ZIRCON trial. As with STARLITE 1 and 2, this trial utilizes ^177^Lu, a β-particle-emitting radionuclide [33].

## 5. Conclusions

In conclusion, girentuximab PET-CT is a novel molecular imaging modality with outstanding diagnostic performance for the diagnosis of ccRCC without the need for biopsy; at present its utility has only been adequately studied for the evaluation of renal masses smaller than 7 cm in localized stages, limiting its indications. Future studies will probably open new paths for this technology, including cancer staging (ruling out metastasis) or therapeutic (combined with lutetium).

## Figures and Tables

**Table 1:** Diagnostic performance of [^89^Zr]Zr-girentuximab PET-CT reported in the ZIRCON clinical trial, overall and according to renal mass size cut-offs.

	Overall (*n* = 284)	≤4 cm (*n* = 145)	≤3 cm (*n* = 76)	<2 cm (*n* = 20)
**Sensitivity**	85.5%	85%	84.4%	96.7%
**Specificity**	87%	89.5%	90.8%	96.7%
Positive predictive value *	92.9%	93.2%	93.7%	96.7%
Negative predictive value *	75.2%	78%	78.2%	96.7%
**Precision**	86%	86.7%	86.8%	96.7%

* Predictive values were calculated with a prevalence of 66% of tumors being clear-cell (182/274); 10 patients were excluded from the table because their SUVs were calculated using DICOM according to the authors; information available in the supplement material of the original publication.

**Table 2:** Summary of diagnostic performance of different imaging modalities for the assessment of small renal masses.

Imaging Modality	Biological Basis	Sensitivity	Specificity	Advantages and/or Pitfalls
**Girentuximab PET-CT**	CAIX expression via VHL pathway.	85.5% (overall)	87.0% (overall)	Non-invasive identification of ccRCC. Hepatobiliary clearance avoids urinary interference.
**Conventional CT Scan**	Anatomical features and enhancement patterns.	~88%	~75%	Initial characterization and staging. Cannot reliably distinguish malignant from benign.
**MRI**	Anatomical features and enhancement patterns.	80–88%	89–90%	Useful for assessing venous invasion and clarifying indeterminate SRMs found on CT.
**FDG PET-CT**	Glucose metabolism.	46–60% (localized); 86–94% (metastatic)	66–100% (localized); 88–100% (metastatic)	Useful for detection of distant osseous and pulmonary metastases. Poor for primary staging due to renal excretion.
**Sestamibi**	Mitochondrial density.	89–92% (for oncocytic lesions)	88–91% (overall); 98% for excluding ccRCC/pRCC	Useful for differentiating benign oncocytic lesions (“hot”) from aggressive ccRCCs (“cold”).
**Contrast-Enhanced Ultrasound (CEUS)**	Microvascular perfusion and enhancement characteristics.	88–100%	80–97%	Improved Bosniak classification and useful in patients with renal impairment.

PET-CT = positron emission tomography-computed tomography; CAIX = carbonic anhydrase IX; VHL = Von Hippel–Lindau; ccRCC = clear-cell renal cell carcinoma; CT = computed tomography; MRI = Magnetic Resonance Imaging; SRM = small renal mass; FDG = fluorodeoxyglucose; pRCC = papillary renal cell carcinoma.

## Data Availability

No new data were created or analyzed in this study. Data sharing is not applicable to this article.
